# A Large Mullerian Duct Cyst Presenting as an Abdominal Mass With Ipsilateral Renal Agenesis: An Unusual Presentation

**DOI:** 10.5812/numonthly.4566

**Published:** 2012-09-24

**Authors:** Shishir Devaraju, Rajendra B. Nerli, Murigendra B. Hiremath

**Affiliations:** 1 Department of Urology, Jawaharlal Nehru Medical College, KLE University, Belgaum, India

**Keywords:** Mullerian Ducts, Cysts, Hereditary Renal Agenesis, Urography

## Abstract

Symptomatic Mullerian duct cysts are uncommon. A young adult male presented to us with a palpable supra-pubic mass, pain and lower urinary tract symptoms. Initial imaging modalities showed a large cystic lesion in the pelvis with a non-visualized right kidney. A short, blind ending right ureter on retrograde pyelography added to the confusion. On exploration, the lesion was noted to be separate from the seminal vesicles, bladder and posterior urethra. The right kidney was absent. The cystic lesion was excised completely preserving the vas and seminal vesicles. A high index of suspicion is needed for identification of this rare condition. Use of MRI (magnetic resonance imaging) can help improve the diagnostic accuracy. Many a times though, the diagnosis is evident only on exploration.

## 1. Introduction

Mullerian duct cyst is a remnant of the fused caudal end of the Mullerian ducts, which normally regresses in utero. mullerian duct cysts associated with symptoms and ipsilateral renal dysgenesis/agenesis is unusual. We herein present a case of an adult male with a large, palpable mullerian duct cyst posing a diagnostic dilemma.

## 2. Case

**Figure 1:**
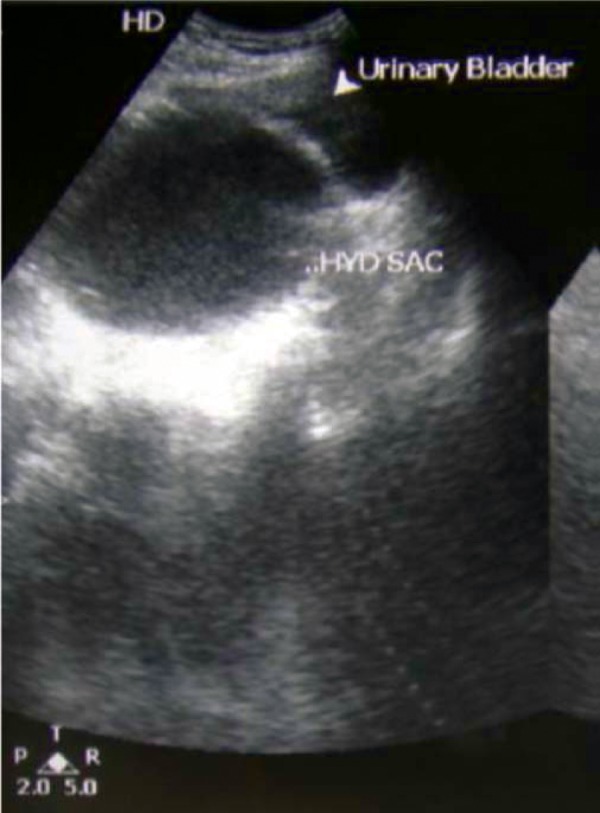
Cystic Lesion Posterior to the Bladder on Ultrasonography

A 28 year old male presented with complaints of pain in the lower abdomen, dysuria, frequency and a swelling in the suprapubic region for the last 2 months. Examination revealed a smooth, firm swelling measuring about 10 × 8 cm in the suprapubic region extending into the right iliac fossa. On per-rectal exam, a smooth bulge was palpable in the anterior wall of the rectum above the prostate. An ultrasound exam of the abdomen showed a large cystic lesion posterior to the bladder with a non visualized right kidney (Figure 1). The left kidney was noted to be normal.

**Figure 2:**
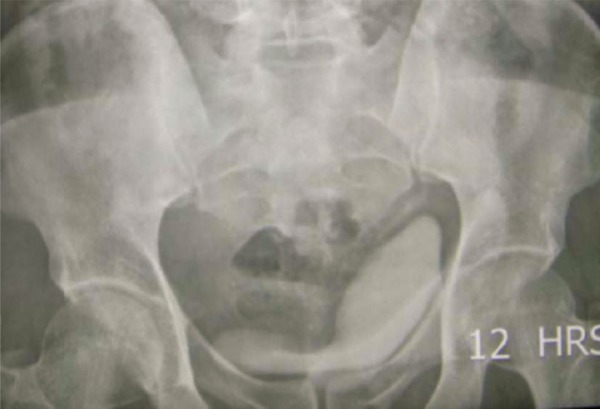
Intravenous Urogram Showing Indentation of Bladder By the Lesion in Delayed Images

**Figure 3:**
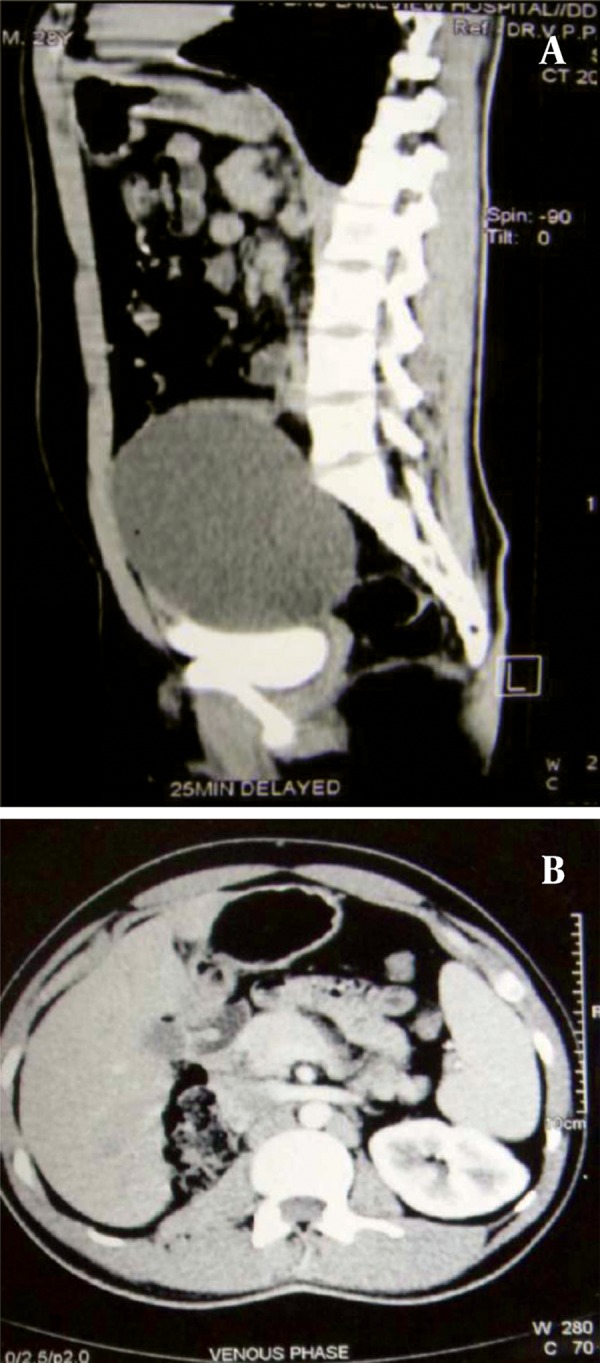
Computerized Tomography Showing the Large Cystic Lesion Above the Bladder (A) and Rt Renal Agensis (B)

**Figure 4:**
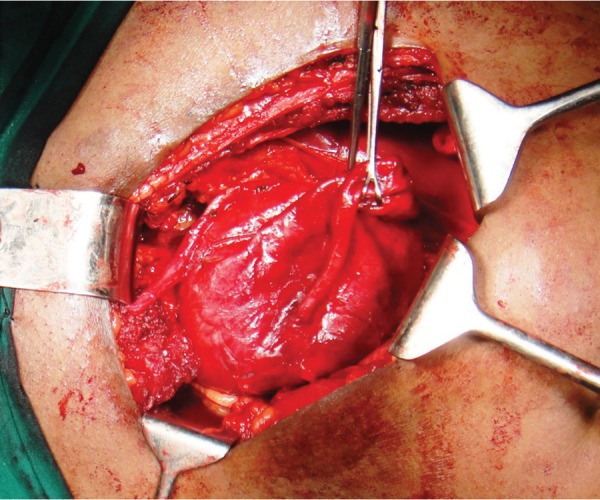
Intra-op Findings

The patient was subjected to an Intravenous Urogram (Figure 2), which showed a normally positioned and functioning left kidney. The right kidney was not visualized even on delayed images. The bladder was noted to be indented on its superior and right postero-lateral aspects. A CT (computed tomography) scan was done, which showed a large cystic lesion behind the bladder, with the right kidney and ureter not separately delineated (Figure 3). On Cystoscopy, the urethra was normal. The right ureteric orifice was present and located laterally and inferior to the level of the left ureteric orifice. On right retrograde pyelography, the dye was noted to pass only a few centimeters along the ureter with an abrupt cut-off. Based on the patient’s history and presence of a large cyst in the pelvis and absence of right kidney in the renal fossa, a differential diagnosis of Pelvi-Ureteric Junction obstruction in an ectopic kidney with thinned out parenchyma was considered. The patient was counseled and consent obtained regarding the need for exploration and nephrectomy if needed. Using a Pfannensteil skin incision, the lesion was approached extraperitoneally. The cystic lesion had no connection to the bladder. A short, blind ending ureteral stump was seen with no renal tissue. This effectively ruled out the initial diagnosis of a grossly hydronephrotic ectopic kidney secondary to PUJ obstruction (Figure 4). The cyst was also noted to be separate from the seminal vesicles and no obvious communication was demonstrable with the posterior urethra. The vas was identified and spared. The cyst was removed completely. A small drain was placed in the extraperitoneal space and abdomen closed in layers. The post operative period was uneventful and patient was discharged on the 4th postoperative day. Histopathology showed a cyst wall lined by stratified cubo-columnar epithelium consistent with a mullerian duct cyst.

## 3. Discussion

In the male embryo’s, sexual differentiation is initiated during 7th week of gestation. The sex determining region Y (SRY) protein activates male transcription factors that cause the bipotential cells to differentiate and proliferate into the testis. Sertoli cells of the testis produce Mullerian Inhibiting substance (MIS), which causes regression of the mullerian ducts by the 10th week of gestation. Hence, decreased production of MIS, inappropriate timing of production and MIS receptor defect can all lead to persistence of the mullerian duct. The cranial end of the mullerian duct remains as appendix testis and the caudal end as the prostatic utricle in normal males (1). Some authors have used the terms mullerian duct remnant (MDR) and mullerian duct cyst interchangeably. However, although the two have similar embryological origins, the clinical presentation is often different.

Mullerian duct cysts present at the 3rd to 4th decade, whereas prostatic utricles are common in the 1st to 2nd decades of life. Mullerian duct cysts do not communicate with the prostatic urethra, but are connected to the verumontanum by a thin stalk. Unlike utricular cysts, the Mullerian duct cyst is not typically associated with other congenital abnormalities of the urinary tract. Very rarely these may be associated with ipsilateral renal agenesis (2). > 90% of utricular cysts are associated with hypospadias or disorders of sexual differentiation and 60-70% with bilateral cryptorchidism. Utricle cysts are small, tubular and do not extend above the base of the prostate. mullerian duct cysts are round or oval in configuration, often large and extend well above the base of the prostate (3-5).

A prevalence of 1-5% (3) has been reported for Mullerian duct cysts, but symptomatic presentation is very rare. The clinical presentation is varied, including urinary frequency, urinary urgency, dysuria, urinary obstruction, hematuria, and pelvic pain. A case of malignant transformation in a 15 year boy has been reported5. Other differential diagnosis of deep pelvic cysts in the male includes seminal vesicle cyst (Zinner’s syndrome, a triad of mullerian duct abnormality comprising of unilateral renal agenesis, ipsilateral seminal vesicle cyst, and ejaculatory duct obstruction), ejaculatory duct cyst, prostatic cyst or abscess, urachal cyst, bladder diverticulum, hydatid disease, and intrapelvic neoplasm. The presence of a large cystic lesion and a short ureteral bud with renal agenesis led us to suspect PUJ in an ectopic pelvic kidney in our patient. MRI has been reported to be useful in the diagnosis of mullerian duct cyst by showing signal characterization of the mucus or hemorrhagic cystic component. An MRI in this patient would have helped achieve a proper pre-op diagnosis.

Treatment is indicated in symptomatic individuals and to prevent long-term complications. Both open surgical techniques and minimally invasive approaches have been described to treat mullerian duct remnants. Surgical management of prostatic utricles is challenging due to their deep location in the pelvis and close relation to important surrounding structures. Endoscopic treatment has been limited to unroofing infected and obstructed cysts. Many different open surgical approaches have been described to excise mullerian duct remnants including transperitoneal, retropubic or suprapubic extravesical, transvesical transtrigonal, posterior sagittal transanorectal, anterior sagittal transanorectal, perineal, and posterior pararectal approaches. Recently Laparoscopic and Robot assisted excision has been utilized for excision of mullerian duct cysts and remnants. It offers the advantage of a clear and magnified view of the deep pelvic structures that cannot be obtained with an open approach, enabling precise dissection with less blood loss and reduced incidence of injury to the surrounding structures (vas, ureters, nerves and urethra) (6, 7). However, port placement and instrument manipulation could be challenging in large cysts, which tend to fill the pelvis and extend into the lower abdomen as in the present case.

## 4. Conclusions

Due to the rare nature of these lesions, a high index of suspicion is necessary for diagnosis. Use of advanced imaging modalities like trans-rectal ultrasound and MRI are useful for aiding the identification of mullerian duct cysts and prostatic utricles. Open surgical exploration remains the mainstay of diagnosis in case of confusion. Treatment is aimed at complete excision of the cyst while preserving the important adjacent structures.

## References

[1] Campbell MF, Wein AJ, Kavoussi LR (2007). Campbell-Walsh Urology.

[2] ULU EMK, Başaran C, Dönmez Fy, Güvenç Z, Güner B, Coşkun M (2009). Müllerian duct cyst with ipsilateral renal agenesis: mri findings. Marmara Med J.

[3] Coppens L, Bonnet P, Andrianne R, de Leval J (2002). Adult mullerian duct or utricle cyst: clinical significance and therapeutic management of 65 cases. J Urol.

[4] Desautel MG, Stock J, Hanna MK (1999). Mullerian duct remnants: surgical management and fertility issues. J Urol.

[5] Warmann SW, Vogel M, Wehrmann M, Scheel-Walter HG, Artlich A, Pereira PL (2006). Giant mullerian duct cyst with malignant transformation in 15-year-old boy. Urology.

[6] Luo JH, Chen W, Sun JJ, Xie D, Mo JC, Zhou L (2008). Laparoscopic Management of Mullerian Duct Remnants: Four Case Reports and Review of the Literature. J Androl.

[7] Hong YK, Onal B, Diamond DA, Retik AB, Cendron M, Nguyen HT (2011). Robot-assisted laparoscopic excision of symptomatic retrovesical cysts in boys and young adults. J Urol.

